# Cost and yield considerations when expanding recruitment for genetic studies: the primary open-angle African American glaucoma genetics study

**DOI:** 10.1186/s12874-017-0374-9

**Published:** 2017-07-14

**Authors:** Rebecca Salowe, Laura O’Keefe, Sayaka Merriam, Roy Lee, Naira Khachatryan, Prithvi Sankar, Eydie Miller-Ellis, Amanda Lehman, Victoria Addis, Windell Murphy, Jeffrey Henderer, Maureen Maguire, Joan O’Brien

**Affiliations:** 1 Scheie Eye Institute, University of Pennsylvania, Philadelphia, PA USA; 2 Philadelphia, PA USA; 3 Lewis Katz School of Medicine, Temple University, Philadelphia, PA USA

**Keywords:** Recruitment, Enrollment, Genetic studies, African Americans, African American recruitment, African American enrollment, Outreach, Biobanks, External sites

## Abstract

**Background:**

African Americans have been historically under-represented in genetic studies. More research is needed on effective recruitment strategies for this population, especially on approaches that supplement traditional clinic enrollment. This study evaluates the cost and efficacy of four supplemental recruitment methods employed by the Primary Open-Angle African American Glaucoma Genetics (POAAGG) study.

**Methods:**

After enrolling 2304 patients from University of Pennsylvania ophthalmology clinics, the POAAGG study implemented four new recruitment methods to supplement clinic enrollment. These methods included: 1) outreach in the local community, 2) in-house screening of community members (“in-reach”), 3) expansion to two external sites, and 4) sampling of the Penn Medicine Biobank. The cost per subject was calculated for each method and enrollment among cases, controls, and suspects was reported.

**Results:**

The biobank offered the lowest cost ($5/subject) and highest enrollment yield (*n* = 2073) of the four methods, but provided very few glaucoma cases (*n* = 31). External sites provided 88% of cases recruited from the four methods (*n* = 388; $85/subject), but case enrollment at these sites declined over the next 9 months as the pool of eligible subjects was depleted. Outreach and in-reach screenings of community members were very high cost for low return on enrollment ($569/subject for 102 subjects and $606/subject for 45 subjects, respectively).

**Conclusions:**

The biobank offered the most cost-effective method for control enrollment, while expansion to external sites was necessary to recruit richly phenotyped cases. These recruitment methods helped the POAAGG study to exceed enrollment of the discovery cohort (*n* = 5500) 6 months in advance of the predicated deadline and could be adopted by other large genetic studies seeking to supplement clinic enrollment.

## Background

Many clinical studies have difficulty recruiting large numbers of subjects. Enrollment can be hindered by a variety of factors, such as strict eligibility criteria, patient reluctance to enroll, or overestimation of the pool of eligible subjects (Lasagna’s Law) [1]. In the United States, more than 70% of clinical trials in 2007 were delayed for up to 6 months due to insufficient enrollment [2]. Similarly, more than two-thirds of multicenter trials (*n* = 114) in the United Kingdom from 1994 to 2002 could not recruit the planned number of subjects within the original timeframe [3]. Insufficient enrollment can have serious consequences, including increased resource and staff expenditures, extension of trial length, and delayed availability of benefit to the public [4, 5].

Genetic studies face unique recruitment challenges. The collection of DNA introduces additional concerns for patients, such as potential discrimination, confidentiality breaches, and misuse of information [6–9]. One study found that participation in genetic cohorts was almost 10% lower than in non-genetic cohorts and that the general population has a skeptical outlook on genetic research [10], though these attitudes are in flux and depend on a number of external factors. These challenges are especially pronounced in African Americans, who are less likely than other ethnic groups to consent to genetic studies or allow DNA to be stored [11, 12]. As a result, this population is under-represented in genetic research [7, 13–15]. In order to increase enrollment in genetic studies, particularly among African Americans, more research is needed on recruitment strategies that overcome these obstacles.

In particular, there is a need to examine approaches to supplement traditional clinic enrollment. Clinic recruitment, while offering a large source of patients, inevitably stagnates over time as the pool of eligible subjects grows smaller and smaller. In recent years, studies have turned to supplementary enrollment methods with varying degrees of success. Outreach screenings enabled studies to reach large numbers of patients, but typically did not yield high enrollment numbers [16]. Media advertisements and brochures in public areas were somewhat effective for one study [7], while another group reported a yield of zero patients [17]. Recruitment at external hospitals or clinics helped a variety of genetic and non-genetic studies to increase enrollment numbers, but sampling across geographic regions can increase the administrative burden of studies and possibly enhance genetic variation [18]. Lastly, DNA samples from biobank populations rapidly increased enrollment numbers for genetic studies [19–21], but these samples often lack the phenotypic information needed to establish robust phenotype-genotype correlations [19]. More research is need on the cost and effectiveness of these methods so they can be successfully applied to future genetic studies.

We addressed this need by investigating supplementary recruitment strategies employed by the Primary Open-Angle African American Glaucoma Genetics (POAAGG) study. The POAAGG study is a 5-year project investigating the genetic risk factors for primary open-angle glaucoma (POAG) in African Americans. Initially, POAAGG subjects were identified solely from three University of Pennsylvania (UPenn) sites, with 2304 patients recruited at these sites before funding from the National Eye Institute (NEI). However, after a couple years of very successful enrollment, recruitment began to slow as the number of eligible glaucoma cases shrank. In order to meet the enrollment goal for the initial discovery cohort (*n* = 5500), the study implemented four new recruitment methods: (1) outreach in the local community, (2) in-house screening of community members (“in-reach”), (3) expansion to external sites, and (4) sampling of the Penn Medicine Biobank (PMBB). The objective of this article is to analyze the enrollment yield, cost, and advantages/disadvantages for each recruitment method, with the goal of identifying the most effective enrollment strategies for the remainder of the POAAGG study and other large genetic studies.

## Methods

### Study population

The POAAGG study population consists of self-identified blacks (African Americans, African descent, or African Caribbean), 35 years or older, identified from the Philadelphia region. Certified clinical research coordinators (CRCs) screened potential subjects based on IRB-approved inclusion/exclusion criteria and approached eligible patients during regularly scheduled visits to ophthalmologists [22]. Participants were initially identified solely from comprehensive and subspecialty clinics at the Scheie Eye Institute, Perelman Center for Advanced Medicine, and Mercy Fitzgerald Hospital (Scheie Eye Institute/UPenn satellite). All enrolled subjects provided a signed informed consent and genomic DNA, which was extracted from peripheral blood or saliva. Glaucoma specialists classified subjects as cases, controls, or suspects (suspected cases) based on previously published criteria [22].

The enrollment goal of the POAAGG study is 7765 subjects, consisting of a discovery cohort of 5500 subjects (2000 cases and 3500 controls) and a validation cohort of 2265 subjects (1000 cases and 1265 controls). Enrollment for the study began in July 2010 and NEI funding was received in March 2014. Beginning in August 2014, the study implemented four new recruitment methods to supplement enrollment from UPenn sites and meet the enrollment goal for the discovery cohort. These methods are detailed below.

### Recruitment method #1: Community outreach

In August 2014, the POAAGG study began to provide comprehensive glaucoma examinations to at-risk members of the Philadelphia community. A mobile van was purchased using funds from an UPenn Hospital Board of Women Visitors grant and was fully equipped with glaucoma screening equipment. The majority of this equipment was borrowed from the Scheie Eye Institute, including a Zeiss Humphrey Frequency Doubling Technology perimeter (Carl Zeiss Meditec, Inc., Dublin, CA), a portable slit lamp (KOWA, Nagoya, Japan), and an ultrasound pachymeter (Reichert, Buffalo, NY). A Cirrus HD-Optic Coherence Tomography (Carl Zeiss Meditec, Inc., Dublin, CA) was leased for 3 years. Additional equipment such as exam chairs, patient privacy screens, and tables were purchased using a Penn CAREs grant. A glaucoma specialist and team of CRCs transported this equipment to community centers, federally qualified health centers, and retirement communities to provide free glaucoma screenings to interested community members. These sites were chosen by directly contacting local organizations or receiving requests from organizations aware of the POAAGG study. Events at more private venues (such as retirement homes) were advertised internally, while screenings at more public sites (such as churches) were announced beforehand and open to the general public. Eligible patients were invited to join the POAAGG study and were classified as case, control, or suspect by a glaucoma specialist. These screenings were also used to capture and refer systemic conditions with ocular manifestations such as diabetes, atherosclerosis, and hypertension.

### Recruitment method #2: In-reach screenings

In October 2014, the POAAGG study began to offer free glaucoma screenings in the Scheie Eye Institute (“in-reach”). Subjects were recruited through publicity in the local community, including a series of advertisements in the local subway (SEPTA), announcements on African American talk radio and newspapers, and outreach through African American pastors and government leaders. Interested community members were encouraged to call CRCs to schedule screenings. Screenings included a comprehensive health history, visual field testing, stereo fundus photography, optic disk OCT imaging, ultrasound pachymetry, and Goldmann applanation tonometry. All eligible patients were enrolled in the POAAGG study and classified as a case, control, or suspect by the glaucoma specialist.

### Recruitment method #3: Expansion to external sites

The POAAGG study expanded enrollment to two external sites in Philadelphia: the private practice of a Scheie Alumnus (Windell Murphy, MD) in West Philadelphia and the Ophthalmology Department at the Lewis Katz School of Medicine at Temple University (Jeffrey Henderer, MD). The sites were chosen because of their proximity to the Scheie Eye Institute, their general patient population (African American and glaucoma patients), and the willingness of the physicians to collaborate on the study. POAAGG recruitment at these sites began in May and July 2015, respectively. CRCs were on-site on specific days and approached eligible patients as they waited to be examined. Subjects gave informed written consent, provided DNA samples, and completed enrollment using the same methods as at UPenn sites. The Director of the Glaucoma Service at UPenn independently checked the status of each suspect enrolled at an external site to ensure this corresponded with classifications defined by the POAAGG study.

### Recruitment method #4: Penn medicine biobank

PMBB is a newly established biobank at UPenn that has enrolled approximately 40,000 patients to date and increases enrollment by approximately 350 patients per week. All patients within UPenn were eligible for voluntary participation in PMBB, which included blood draw for DNA extraction, tissue sampling (if applicable), and a questionnaire. All PMBB subjects have consented to have their DNA used for research studies at UPenn.

PMBB subjects eligible to participate in the POAAGG study were mailed a letter providing the opportunity to opt-out of the study. In September 2015, PMBB provided de-identified aliquots of DNA from 2073 eligible African American subjects, along with information on gender and ICD-9 codes relevant to glaucoma, permitting an initial classification of DNAs as likely-case or likely-control. These subjects were added to the POAAGG discovery cohort, which was subsequently genotyped using the Illumina Infinium Multi Ethnic Genotyping Array (MEGA).

Several approaches were used to obtain phenotypic information on PMBB subjects. All PMBB patients were mailed a brochure that explained the study and provided a tear-off page to mail back with glaucoma status and family history information. A subset of patients were previously seen at the UPenn Ophthalmology Department (*n* = 583), which provided a definitive classification as case, control, or suspect, as well as phenotypic information. We attempted to sample the remainder of PMBB subjects in an effort to obtain an accurate estimate of undiagnosed glaucoma in the likely-control group in the following manner. Biostatisticians chose a random sample of 125 subjects with the same age structure as the previously defined PMBB control group. These subjects were invited to the Scheie Eye Institute for a free glaucoma screening with associated $100 compensation, with the goal of screening a minimum of 100 subjects (80%). Despite our best efforts, however, the recruitment goal of 80% remained unmet due to erroneous contact information, unreturned phone calls, and subjects declining participation. Instead, age-stratified estimates of undiagnosed glaucoma from the NEI [23] and Baltimore Eye Survey [24] will be used to approximate the rate of undiagnosed glaucoma in the PMBB population.

### Cost analysis

The expense breakdown for each recruitment method from the perspective of the POAAGG study is shown in Table 1. Any purchased equipment (even if funded by additional grants) was included in the cost analysis to ensure accurate comparisons. Equipment borrowed from the Scheie Eye Institute for outreach and in-reach screenings, as well as equipment already present at external sites, was not included. No payment was made to obtain DNA samples from PMBB or to recruit from ophthalmologists at external sites. All subjects, regardless of disease status, were counted equally, as all were ultimately genotyped.

**Table 1:** Cost Breakdown for Supplemental Recruitment Methods

*Purchase*	*Cost*
Community Outreach	
Necessary Equipment (Penn CAREs Grant)	$2000.00
Optical Coherence Tomography (lease per year)	$19,392.00
Pamphlets about POAAGG Study	$1195.00
Subject Blood Collection^a^ (26 subjects)	$73.32
Subject Gift Cards† (102 subjects)	$1020.00
Subject Spit Kits‡ (76 subjects)	$1140.00
Personnel Time to Prepare and Conduct Event	$9248.00
Van Purchase	$20,180.00
Van Storage (per year)	$3818.00
*Total (102 enrolled subjects)*	$58,066.32
*Total Per Subject*	$569.28
In-reach Screenings	
Advertisement via SEPTA Ads	$3050.00
Necessary Equipment (Penn CAREs Grant)	$2000.00
Optical Coherence Tomography (lease per year)	$19,392.00
Subject Blood Collection (3 subjects)	$8.46
Subject Gift Cards (45 subjects)	$450.00
Subject Spit Kits (42 subjects)	$630.00
Personnel Time	$1721.25
*Total (45 enrolled subjects)*	*$27,251.71*
*Total Per Subject*	*$605.59*
Expansion to External Sites	
Subject Gift Cards (850 subjects)	$8500.00
Subject Spit Kits	$12,750.00
POAAGG Personnel Time at Private Practice	$45,344.00
POAAGG Personnel Time at Temple	$9384.00
POAAGG Physician Time for Subject Reclassifiction	$4666.61
Staff Transportation to Private Practice	$1908.00
Staff Transportation to Temple	$400.50
*Total (850 enrolled subjects)*	*$82,953.11*
*Total Per Subject*	*$84.82*
PMBB	
Mailing of Opt-Out Cards	$721.70
Mailing of POAAGG Brochures	$4159.00
Personnel Time for Review of PMBB Patient Records	$4511.04
*Total (2073 enrolled subjects)*	*$9391.74*
*Total Per Subject*	*$4.53*

^a^Blood Collection: $2.82 /subject; †Gift cards: $10/subject; ‡ Spit Kits: $15/subject

## Results

The POAAGG study has enrolled 7959 subjects as of 12/01/2016, including 2423 cases, 4376 controls, and 1160 suspects. The four supplemental recruitment methods were instituted in August 2014 (community outreach), October 2014 (in-reach screenings), May and July 2015 (two new external sites), and September 2015 (PMBB).

**Fig. 1:**
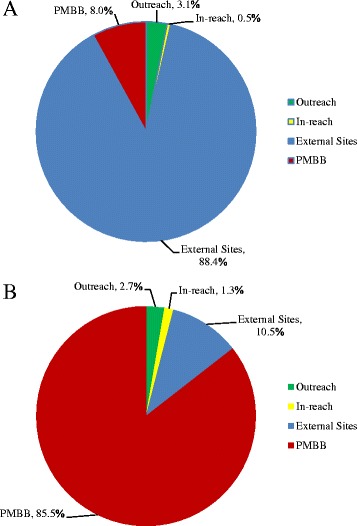
Sources of **a** case enrollment from supplemental recruitment methods (*n* = 388) and **b** control enrollment from supplemental recruitment methods (*n* = 2285). *Abbreviation*: *PMBB* Penn Medicine Biobank

**Fig. 2:**
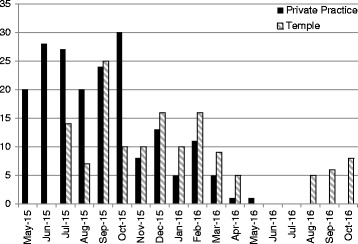
Case enrollment at two external sites over time, including private practice and Temple University

PMBB sampling resulted in the highest enrollment numbers (*n* = 2073), followed by external sites (*n* = 850), community outreach (*n* = 102), and in-reach screenings (*n* = 45) (Table 2). Cases constituted 40% of total enrollment from external sites, but made up a smaller proportion of enrollment from community outreach (12%), in-reach screenings (4%), and PMBB sampling (1%). The majority of cases were enrolled from external sites (88%) and the majority of controls were enrolled from PMBB (86%) (Fig. 1). Monthly case enrollment at both external sites declined over the course of a year (Fig. 2).

**Table 2:** Subject Enrollment and Costs from Supplemental Recruitment Methods

	Enrollment				Costs	
Site	Cases	Controls	Suspects	Total	Total Cost	Cost/Patient
Community Outreach	12	62	28	102	$58,066.32	$569.28
In-reach Screening	2	30	13	45	$27,251.71	$605.59
External Sites	343	239	268	850	$82,953.11	$84.82
PMBB	31	1954	88	2073	$9391.74	$4.53

PMBB sampling had the lowest cost per subject ($5/subject), followed by external sites ($85/subject), community outreach ($569/subject), and in-reach screening ($606/subject). Overall, community outreach and in-reach screenings were high cost and low yield ($58,066.32 for 102 subjects and $27,251.71 for 45 subjects, respectively). External sites were moderate cost with strong enrollment, particularly among cases ($82,953.11 for 850 subjects, including 343 cases). PMBB was low cost with very high total enrollment ($9391.74 for 2073 subjects).

## Discussion

This study contrasted the cost and efficacy of four methods to supplement traditional enrollment for the POAAGG study. We found that biobanks offered the most cost-effective method for subject enrollment, while expansion to external sites was necessary to recruit richly phenotyped cases.

Community-based events provided opportunities to screen at-risk members of the Philadelphia community and refer individuals with systemic diseases. However, from a purely recruitment perspective, these screenings were extremely expensive for a low return on enrollment. The majority of costs for outreach events arose from equipment expenses and personnel time, as a team of eight CRCs devoted approximately 12 h to each event. A significant portion of time was also spent screening ineligible patients, with 82 (44.3%) out of 185 screened patients at outreach events not qualifying for the POAAGG study. In the future, the POAAGG study will continue to accept eligible subjects identified during outreach events, but the events are now mainly considered a public service, rather than a recruitment method. We have discontinued in-reach due to its high cost and extremely low yield.

Recruitment from external sites was more expensive than PMBB, but significantly more effective for case enrollment. The majority of expenses arose from subject spit kits, subject gift cards, and personnel time—all normal enrollment costs at UPenn sites. The community ophthalmologists were extremely generous to open their clinics free of charge, viewing the arrangement as a research collaboration with a realized potential to publish results together and serve the Philadelphia community. Unlike outreach, in-reach, and PMBB subjects, cases from external sites had medical history and phenotypic information readily available and presented the most predictable and reliable base for case enrollment. However, enrollment at external sites eventually encountered the same saturation problem as UPenn sites. The sites were able to sustain enrollment for the span of 6 to 9 months, before the costs associated with dispatching staff to these sites outweighed the number of new cases recruited.

In contrast, PMBB was the least expensive recruitment method and rapidly increased enrollment numbers. A large reason that costs were so low was that researchers at PMBB generously provided all DNA free of cost. Once PMBB becomes more established, it will likely be necessary to charge a fee per sample for future studies. Fees for biobank samples range greatly, with an international expert group recommending prices between $10 and $100 per sample for a marginal-cost model [25]. With such pricing, biobanks samples could become just as expensive as enrolling a fully phenotyped patient at an external site. This will be important to consider, as biobank patients are not phenotyped and often do not have a definitive disease status. POAAGG researchers were able to address the phenotypic information issue in a subset of PMBB patients that were previously seen at the UPenn Ophthalmology Department and had complete electronic medical records (*n* = 583). Twenty-five of the 583 patients (4.3%) were re-classified as glaucoma cases, which is very close to prevalence estimates of glaucoma from the NEI (4.2%) [23] and Baltimore Eye Survey (5.0%) [24]. The remainder of PMBB subjects was not seen by the Ophthalmology Department and has been classified as PMBB controls. A subset of these patients likely has undiagnosed glaucoma, which will be accounted for in the genetic analyses using the estimates described above. Researchers also have access to PennSeek, an UPenn developed Google-type search engine for the electronic medical record, which will allow further characterization of PMBB subjects as the study proceeds.

The four recruitment methods outlined allowed the POAAGG study to add 2970 additional patients to its cohort, exceeding enrollment of the discovery cohort (*n* = 5500) 6 months in advance of the proposed deadline. Based on the findings of this paper, the POAAGG study plans to take the following steps to complete (and possibly exceed) enrollment for the validation cohort (*n* = 2265): 1) Obtain a new sample of subjects from PMBB to build enrollment in the control group; 2) Begin focused case recruitment at two new external sites in the Philadelphia region, Drexel University and UPenn-affiliated Chester County Hospital; 3) Continue traditional clinic enrollment at existing UPenn sites to obtain phenotyped controls and a small number of new cases; and 4) Continue outreach events to provide glaucoma screenings to at-risk populations without relying upon these events to boost enrollment numbers.

We recommend that investigators of large genetic studies consider implementing these methods if clinic enrollment declines over time. Biobanks can provide a low-cost, high-yield source of control subjects, while external sites can yield high numbers of phenotyped subjects with the disease of interest. Expansion to more than one external site may be necessary to combat stagnation in enrollment, as was demonstrated in this study. We found that each new site yielded steady enrollment for approximately 9 months, since most glaucoma cases and high-risk suspects are evaluated every 3 to 6 months. These results suggest that studies can anticipate a drop off in enrollment numbers after a cycle of two to three patient appointments.

Limitations of this study include the estimation of several variables for cost calculations. Exact numbers were used when available (i.e. purchase of the mobile van), but it was necessary to approximate other costs (i.e. personnel time before, during, and after outreach events). Our results also may be constrained by specifics related to the POAAGG study and Philadelphia region. The study exclusively enrolls patients of African descent, possibly compromising the generalizability of our findings to other racial and ethnic groups. In addition, because the POAAGG study is a case-control study and not treatment-based, it does not require any follow-up visits. Thus, our recommendations may not fully reflect the challenges faced by other recruiters.

## Conclusions

Genetic studies often have difficulty reaching high enrollment targets, especially when recruiting African American patients [11, 12]. Even when recruitment from hospital clinics is successful, enrollment tends to decline over time as the pool of eligible patients shrinks. The POAAGG study encountered this “saturation” problem after successfully recruiting more than 2000 patients from UPenn clinics. This research closely examines our resultant efforts to supplement enrollment with four new strategies. We found that community-based screenings, while allowing very worthwhile screening of at-risk community members, were extremely expensive from a recruitment perspective for a low return on enrollment. In contrast, the biobank offered rapid increases in control numbers and external sites provided richly phenotyped cases.

## Abbreviations

CRC: Clinical research coordinator
MEGA: Multi ethnic genotyping array
NEI: National Eye Institute
PMBB: Penn Medicine Biobank
POAAGG: Primary Open-Angle African American Glaucoma Genetics Study
POAG: Primary open-angle glaucoma
UPenn: University of Pennsylvania

## References

[1] Lasagna L (1979). Problems in publication of clinical trial methodology. Clin Pharmacol Ther.

[2] Roan S. Medical Clinical Research Slows for Lack of Patients, L.A. Times. 2009. Available at http://articles.latimes.com/2009/mar/14/science/sci-clinical-trials14.

[3] Campbell MK, Snowdon C, Francis D, Elbourne D, McDonald AM, Knight R, Entwistle V, Garcia J, Roberts I, Grant A (2007). Recruitment to randomised trials: strategies for trial enrollment and participation study. The STEPS study. Health Technol Assess.

[4] Watson JM, Torgerson DJ (2006). Increasing recruitment to randomised trials: a review of randomised controlled trials. BMC Med Res Methodol.

[5] Thoma A, Farrokhyar F, McKnight L, Bhandari M (2010). How to optimize patient recruitment. Can J Surg.

[6] Bogner HR, Wittink MN, Merz JF, Straton JB, Cronholm PF, Rabins PV, Gallo JJ (2004). Personal characteristics of older primary care patients who provide a buccal swab for apolipoprotein E testing and banking of genetic material: the spectrum study. Community Genet.

[7] Royal C, Baffoe-Bonnie A, Kittles R, Powell I, Bennett J, Hoke G, Pettaway C, Weinrich S, Vijayakumar S, Ahaghotu C (2000). Recruitment experience in the first phase of the African American hereditary prostate cancer (AAHPC) study. Ann Epidemiol.

[8] Hughes GD, Sellers DB, Fraser LB, Knight B, Areghan GA (2003). Barriers and strategies for sustained participation of African-American men in cohort studies. Ethn Dis.

[9] Schulz A, Caldwell C, Foster S (2003). “what are they going to do with the information?” Latino/Latina and African American perspectives on the human genome project. Health Educ Behav.

[10] Matsui K, Kita Y, Ueshima H (2005). Informed consent, participation in, and withdrawal from a population based cohort study involving genetic analysis. J Med Ethics.

[11] Sterling R, Henderson GE, Corbie-Smith G (2006). Public willingness to participate in and public opinions about genetic variation research: a review of the literature. Am J Public Health.

[12] Lee SS, Mountain J, Koenig BA (2001). The meanings of “race” in the new genomics: implications for health disparities research. Yale J Health Policy Law Ethics.

[13] Moorman PG, Skinner CS, Evans JP, Newman B, Sorenson JR, Calingaert B, Susswein L, Crankshaw TS, Hoyo C, Schildkraut JM (2004). Racial differences in enrolment in a cancer genetics registry. Cancer Epidemiol Biomark Prev.

[14] Armstrong K, Micco E, Carney A, Stopfer J, Putt M (2005). Racial differences in the use of BRCA1/2 testing among women with a family history of breast or ovarian cancer. JAMA.

[15] Peprah E, Xu H, Tekola-Ayele F, Royal CD (2015). Genome-wide association studies in Africans and African Americans: expanding the framework of the genomics of human traits and disease. Public Health Genomics.

[16] Victor RG, Haley RW, Willett DL, Peshock RM, Vaeth PC, Leonard D, Basit M, Cooper RS, Iannacchione VG, Visscher WA (2004). The Dallas heart study: a population-based probability sample for the multidisciplinary study of ethnic differences in cardiovascular health. Am J Cardiol.

[17] Patterson AR, Davis H, Shelby K, McCoy J, Robinson LD, Rao SK, Banerji P, Tomlinson GE (2008). Successful strategies for increasing African American participation in cancer genetic studies: hopeful signs for equalizing the benefits of genetic medicine. Community Genet.

[18] DE BK, Macpherson M, Reich D, Mountain JL (2015). The genetic ancestry of African Americans, Latinos, and European Americans across the United States. AJHG.

[19] Founti P, Topouzis F, van Koolwijk L, Traverso CE, Pfeiffer N, Viswanathan AC (2009). Biobanks and the importance of detailed phenotyping: a case study--the European glaucoma society GlaucoGENE project. Br J Ophthalmol.

[20] Elmore JR, Obmann MA, Kuivaniemi H, Tromp G, Gerhard GS, Franklin DP, Boddy AM, Carey DJ (2009). Identification of a genetic variant associated with abdominal aortic aneurysms on chromosome 3p12.3 by genome wide association. J Vasc Surg.

[21] Amirian ES, Armstrong GN, Zhou R, Lau CC, Claus EB, Barnholtz-Sloan JS, Il’yasova D, Schildkraut J, Ali-Osman F, Sadetzki S (2016). The Glioma international case-control study: a report from the genetic epidemiology of Glioma international consortium. Am J Epidemiol.

[22] Charlson E, Sankar P, Miller-Ellis E, Regina M, Fertig R, Salinas J, Pistelli M, Salowe R, Rhodes A, III WM (2015). The primary open-angle African-American glaucoma genetics (POAAGG) study: baseline demographics. Ophthalmology.

[23] USA, National Eye Institute. Glaucoma, Open-angle. 2010.

[24] Tielsch JM, Sommer A (1991). Racial variation in the prevalence of primary open angle glaucoma: the Baltimore eye survey. JAMA.

[25] Clement B, Yuille M, Zaltoukal K, Wichmann HE, Anton G, Parodi B, Kozera L, Brechot C, Hofman P, Dagher G (2014). Public biobanks: calculation and recovery of costs. Sci Transl Med.

